# Objective, Clinician- and Patient-Reported Evaluation of Late Toxicity Following Adjuvant Radiation for Early Breast Cancer: Long-Term Follow-Up Results of a Randomised Series

**DOI:** 10.3390/jcm12134212

**Published:** 2023-06-22

**Authors:** Cas Stefaan Dejonckheere, Alina Abramian, Kira Lindner, Anne Bachmann, Katharina Layer, Teresa Anzböck, Julian Philipp Layer, Gustavo Renato Sarria, Davide Scafa, David Koch, Christina Leitzen, Christina Kaiser, Andree Faridi, Leonard Christopher Schmeel

**Affiliations:** 1 Department of Radiation Oncology, University Hospital Bonn, 53127 Bonn, Germany; cas.dejonckheere@ukbonn.de (C.S.D.); katharina.layer@ukbonn.de (K.L.);; 2 Department of Gynaecology, Division of Senology, University Hospital Bonn, 53127 Bonn, Germanyandree.faridi@ukbonn.de (A.F.); 3 Department of Gynaecology, Division of Gynaecology and Gynaecological Oncology, University Hospital Bonn, 53127 Bonn, Germany; teresa.anzboeck@ukbonn.de; 4 Institute of Experimental Oncology, University Hospital Bonn, 53127 Bonn, Germany

**Keywords:** breast cancer, radiation therapy, conventional fractionation, moderate hypofractionation, late toxicity, pigmentation changes, fibrosis, spectrophotometry, ultrasound, breast cosmesis

## Abstract

**Background and Purpose:** This study aimed to differentially assess the frequency and severity of late radiation-induced toxicity following adjuvant whole-breast irradiation for early breast cancer with conventional fractionation (CF) and moderate hypofractionation (mHF). **Materials and Methods:** Patients recruited in a previous randomised controlled trial comparing acute toxicity between CF and mHF without disease recurrence were included in a post hoc analysis. Spectrophotometric and ultrasonographic examinations were performed for an objective evaluation and subsequent comparison of long-term skin toxicity. Furthermore, patient- and clinician-reported outcomes were recorded. **Results:** Sixty-four patients with a median age of 58 (37–81) years were included. The median follow-up was 57 (37–73) months. A total of 55% underwent CF and 45% mHF. A total of 52% received a sequential boost to the tumour bed. A significant decrease in mean L* (*p* = 0.011) and an increase in a* (*p* = 0.040) and b* values (*p* < 0.001) were observed, indicating hyperpigmentation. In comparison with the non-irradiated breast, there was a significant increase in both cutis (+14%; *p* < 0.001) and subcutis (+17%; *p* = 0.011) thickness, significantly more pronounced in CF patients (*p* = 0.049). In CF patients only, a sequential boost significantly increased the local cutis thickness and oedema compared to non-boost regions in the same breast (*p* = 0.001 and *p* < 0.001, respectively). **Conclusions:** mHF objectively resulted in reduced long-term skin toxicity compared to CF. A sequential boost increased the local fibrosis rate in CF, but not in mHF. This might explain the subjectively reported better cosmetic outcomes in patients receiving mHF and reinforces the rationale for favouring mHF as the standard of care.

## 1. Introduction

Early breast cancer remains the most common cancer diagnosis worldwide [1]. Treatment usually includes lumpectomy followed by whole-breast irradiation (WBI) to consolidate local control and improve survival [2]. In recent years, moderate hypofractionation (mHF) has been established as a standard of care for WBI; however, its potential benefit over conventional fractionation (CF) regarding late skin toxicity has been investigated by fewer trials [3,4,5,6]. Acute skin toxicity, particularly radiation dermatitis, is frequent and often affects a patient’s quality of life [7,8,9,10]. Nevertheless, it is mostly self-limiting and usually resolves within weeks after completion of the radiation course. Late toxicity, in contrast, can be irreversible and thus generate a long-lasting impairment in quality of life [11]. Frequent late toxicities include pigmentation changes, telangiectasia, and cutaneous and subcutaneous fibrosis, which are known to affect breast cosmesis [12]. If the regional lymph nodes are included in the radiation field, potential additional risks are posed, i.e., arm lymphoedema, reduced shoulder mobility, or plexopathy. Constitutional symptoms, such as long-term fatigue, arise in up to one-third of patients [13,14].

An increasing body of mature data exists on local control and survival with modern radiation techniques or alternative fractionation regimens. Nonetheless, in-depth evidence on late toxicity has not been thoroughly researched. Hurdles include a lack of non-invasive objective assessment methods, poor correlation between clinician- and patient-reported outcomes (CROs; PROs), and the long intervals between irradiation and onset of symptoms, delaying a timely and accurate diagnosis [8,15].

The aim of this study was to comprehensively assess late radiation-induced toxicity in patients who underwent adjuvant WBI after breast-conserving surgery, using both objective and CRO and PRO measures. Furthermore, differences between CF and mHF were evaluated.

## 2. Materials and Methods

### 2.1. Participants

Patients receiving WBI for early breast cancer at our comprehensive university cancer center and other participating clinics between October 2016 and July 2019 were screened for inclusion (*n* = 143). All patients from this collective had been previously recruited into a randomised controlled trial investigating differences in acute radiation-induced toxicity between CF and mHF [4]. Inclusion criteria for this initial trial were age > 18 years, breast-conserving surgery for breast cancer, and intended WBI, either as CF or as mHF. Exclusion criteria were planned irradiation of regional lymph nodes, history of ipsilateral breast irradiation, mastectomy, reconstruction with breast implant, metastatic disease, active dermatitis in the breast region, pre-existing dermatological disorder, current use of corticosteroids, and refusal to participate.

All patients who had completed the initial trial without treatment interruptions were reassessed for inclusion in this post hoc analysis (*n* = 140). To reduce the risk of bias in the assessment of late toxicity, the following exclusion criteria were defined: ipsilateral recurrent disease, metastases, contralateral breast irradiation (since the non-irradiated breast is used as control), reconstruction with breast implant, active dermatitis of the breast, any pre-existing dermatological condition, current use of corticosteroids. Patients meeting the inclusion criteria were contacted and invited to participate in this follow-up examination. Prior to their appointment, all patients received a PRO questionnaire by mail. Written informed consent was obtained from all participants. This study was conducted in accordance with the Declaration of Helsinki, approved by the Institutional Review Board (184/22) and preregistered in the German Clinical Trials Register (DRKS 00029665).

### 2.2. Radiation Protocol

All patients received either 50 Gy in 25 fractions of 2 Gy (CF) or 40.05 Gy in 15 fractions of 2.67 Gy (mHF), using 6 MV sliding window intensity-modulated radiotherapy (IMRT) or hybrid 6 and 10 MV volumetric modulated (partial) arc therapy (VMAT) [4]. A sequential normofractionated boost to the tumour bed (16 Gy in 8 fractions of 2 Gy) was given to patients with positive tumour margins, age ≤ 50 years, and age ≥ 51 in case of a high-grade tumour (≥pT2, HER2/neu positive, triple-negative, poor cell differentiation). The International Commission on Radiation Units and Measurements (ICRU) recommendations for dose limits of 95% to 107% were followed. All patients were treated on a TrueBeam STx (Varian Medical Systems, Palo Alto, CA, USA) linear accelerator in a supine position on a breast board. Left-sided WBI was performed in deep inspiration breath-hold (DIBH), if feasible.

Standard institutional skin care with a urea-based lotion (Eucerin UreaRepair PLUS 5%, Beiersdorf, Hamburg, Germany) was applied twice daily to the whole breast, from the first day of treatment onwards until completion. Patients presenting with grade ≥ 2 radiation dermatitis with moist desquamation and severe pain during radiation treatment, were prescribed topical corticosteroids, until symptoms resolved.

### 2.3. Patient Evaluation

#### 2.3.1. Clinical Examination

Biometric data and patient characteristics (comorbidities, smoking habits, breast volume, prior systemic therapies) were collected. Clinical examination was performed by an experienced breast radiation oncologist, scoring relevant Late Effects of Normal Tissue—Subjective, Objective, Management, and Analytic (LENT-SOMA) items (Table A1) [16,17]. Furthermore, the cosmesis of the irradiated breast relative to the untreated contralateral breast was assessed by an experienced breast surgeon as being excellent, fair, good, or poor, according to the Harvard Breast Cosmesis Scale (Table A2) [18]. Clinicians were blinded to the radiation treatment characteristics (fractionation regimen).

#### 2.3.2. Objective Assessments

**Figure 1:**
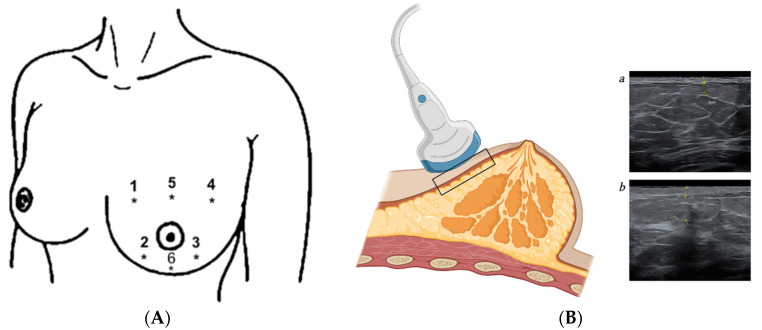
(**A**) Location of the 6 spectrophotometric (1–6) and 4 ultrasound (1–4) measurements. Corresponding ultrasound points were identified and measured on the contralateral breast. (**B**) Quantifying tissue fibrosis using ultrasound. The thickness of the skin (epidermis and dermis), subcutis, and subcutaneous oedema (if present) was measured for the contralateral (**a**) and ipsilateral (**b**) breast. Created with BioRender.com (accessed on 1 March 2023).

Pigmentation changes were assessed using a reflectance spectrophotometer (CR-10 Plus, Konica Minolta, Tokyo, Japan). Six readings were performed on the irradiated breast (Figure 1A). This compact device has been previously used to assess pigmentation changes in a non-invasive and objective way [4,19,20,21]. The automatically performed measurements are based on the Commission Internationale de l’Éclairage (CIE) system of tristimulus values, describing each measured colour (or skin tone) with three coordinates, using the L*a*b* system. The L* value describes the luminance or brightness of the skin, the a* value describes the position of the colour on a scale ranging from red to green, and the b* value describes the position on a scale from blue to yellow. Accordingly, higher L* values describe lighter skin, whereas lower values indicate hyperpigmentation. Higher a* values indicate erythema. Since all patients had been previously enrolled in a randomised controlled trial, baseline spectrophotometric measurements (before radiation treatment initiation) were available.

The extent of tissue fibrosis was quantified using ultrasound, which has previously proved reliable and valid in this context [22,23]. Patients were in a supine position with the axis of the ultrasound probe perpendicular to the skin surface, applying minimal pressure. To guarantee proper coupling between the patient’s skin and the probe, a thin layer of transmission gel was used. All breast examinations were performed in B-mode, using 4–13 MHz ML6-15-D linear array probes on a Voluson E10 (GE Healthcare, Solingen, Germany). The thickness of the skin (epidermis and dermis) and subcutis were measured in each of the breast quadrants (1, 4, 8, and 10 o’clock) of both breasts (Figure 1A,B). In patients who received a sequential boost to the tumour bed, the skin and subcutis thickness in this region were documented separately, using the surgical scar and the original digital radiation treatment plan to determine the exact location. Anatomically, the transition between cutis and subcutis can be determined very precisely, as the cutis consists mainly of keratinocytes, whereas the subcutis is connective and adipose tissue, reflected by a different image morphology in the ultrasound. If subcutaneous oedema was present, this was documented and quantified by ultrasound as well. A single senior breast surgeon with certified expertise in breast ultrasound performed all measurements to avoid interobserver variability. To minimise any potential bias, this physician was blinded to the radiation treatment characteristics (fractionation regimen), as well as the corresponding clinically assessed late toxicity.

#### 2.3.3. Patient-Reported Outcome

Items such as appetite, fatigue, itching, pain, self-image, sexual health, and satisfaction with breast cosmesis were subjectively evaluated using a modified Patient-Reported Outcomes of the Common Terminology Criteria for Adverse Events (PRO-CTCAE) questionnaire (Table A3) [24]. The severity of each item and its influence on activities of daily living and quality of life were scored on a 5-point Likert scale, separately for the ipsilateral and contralateral breast.

### 2.4. Endpoints and Statistical Analysis

The patients recruited for this study had already participated in a prospective clinical trial on the acute toxicity of CF versus mHF WBI [4]. We were thus able to draw from a collective previously homogenised by means of randomisation and stratification. The primary endpoint was the incidence of late radiation-induced breast fibrosis, as measured by ultrasonographic skin thickness in both treatment arms.

Mean, median, standard deviation (SD), and range were calculated for all applicable clinical data. Differences in baseline patient characteristics by randomisation arm were assessed using Pearson’s *χ^2^* or the Wilcoxon rank-sum test (if the data were not normally distributed), as appropriate. The comparison of spectrophotometric and ultrasonographic data by randomisation arm was performed with an unpaired two-sample *t*-test, after checking homoscedasticity using Levene’s test. To compare with baseline or contralateral values, a paired *t*-test was used. For the comparison of clinician- and patient-assessed scores (categorical variables) among both fractionation regimens or depending on the presence or absence of a sequential boost, Pearson’s *χ*^2^ was calculated. If a correlation between CRO or PRO and a continuous variable (i.e., spectrophotometric or ultrasonographic values) was sought, an ANOVA was performed, and correlation was quantified using Pearson’s *r*. Finally, concordance between CRO and PRO was quantified with Cohen’s *κ*, using the reference values as proposed by Landis and Koch [25]. The statistical significance level was defined as *p* < 0.05, using SPSS Statistics version 27 (IBM, Armonk, New York, USA) to perform the analyses.

## 3. Results

### 3.1. Patient and Treatment Characteristics

**Figure 2:**
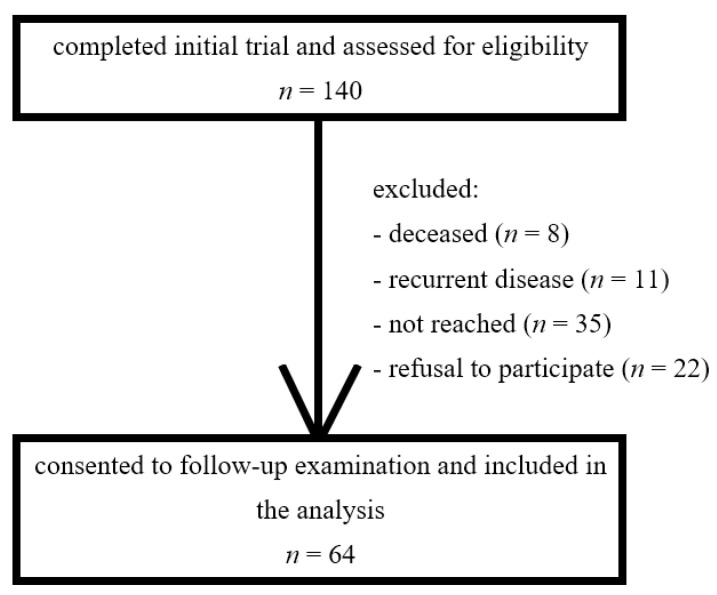
Flowchart of patient selection and inclusion. Patients completing the initial trial [4] were reassessed for eligibility in this follow-up trial. Exclusion criteria were defined as ipsilateral recurrent disease, metastases, contralateral breast irradiation, reconstruction with breast implant, active dermatitis of the breast, any pre-existing dermatological condition, and current use of corticosteroids.

The assessments were completed between September 2022 and February 2023. In total, 64 patients (45.7% of the initial cohort) consented to the follow-up examination and were included in the analysis (Figure 2). All patients were female and Caucasian. Their median age (range) was 58 (37–81) years, and the median time between radiation treatment completion and late toxicity assessment was 57 (37–73) months. A total of 54.7% underwent CF and 45.3% mHF. A total of 51.6% had received a sequential boost to the tumour bed. A total of 6.3% of patients received VMAT, and all other patients underwent IMRT. Patient and treatment characteristics were well balanced between both arms and are summarised in Table 1.

### 3.2. Objective Assessments

#### 3.2.1. Pigmentation Changes

A total of 384 spectrophotometric readings were performed. Compared to baseline skin tone measurements, there was a significant decrease in the mean L* value (*p* = 0.011), indicating darker skin or hyperpigmentation. Furthermore, a significant increase in the mean a* value (*p* = 0.040) and mean b* value (*p* < 0.001) was noted, translating into more red and yellow tones, respectively, but no significant changes regarding the mean differences in the spectrophotometric skin measurements between the two fractionation groups: *p* = 0.281, *p* = 0.076, and *p* = 0.559 for L*, a*, and b*, respectively. Table 2 summarises the spectrophotometry data.

#### 3.2.2. Tissue Fibrosis

A total of 545 ultrasonographic measurements were conducted. There was a significant increase in both cutis (+14.1%) and subcutis (+17.1%) thickness when compared to the contralateral, non-irradiated breast (*p* < 0.001 and *p* = 0.011, respectively). A total of 17.5% of patients had measurable subcutaneous oedema. The increase in cutis thickness was significantly more pronounced in patients who had previously undergone a conventionally fractionated radiation regimen (*p* = 0.049). For the subcutis, this difference was not significant (*p* = 0.088), but for the combined cutis and subcutis thickness, the effect remained significant (*p* = 0.047). Regarding oedema, there were no differences in the prevalence or extent between both treatment arms (*p* = 0.332) (Table 3).

When comparing patients with and without a sequential boost to the tumour bed, there was no significant increase in the overall cutis or subcutis thickness (*p* = 0.430 and *p* = 0.555, respectively). However, when comparing boost regions to non-boost regions in the same breasts, there was a significant increase in cutis (*p* < 0.001), combined cutis and subcutis (*p* = 0.006), and oedema (*p* = 0.015) thickness. Considering only patients receiving CF, these increases in the boost region remained significant (*p* = 0.001, *p* = 0.026, and *p* < 0.001, respectively). However, this was not the case in patients who underwent mHF (*p* = 0.209, *p* = 0.099, and *p* = 0.817, respectively) (Table 4).

### 3.3. Clinician-Reported Outcome

None of the mean LENT-SOMA item scores was significantly different between the two fractionation arms (Table 5). The blinded cosmesis assessment was significantly better in patients who underwent mHF (*p* = 0.024) (Table 6). In patients who had previously received a sequential boost to the tumour bed, there was increased clinician-assessed pain (*p* = 0.006) and fibrosis (*p* = 0.070). The blinded cosmesis assessment was not influenced by boost application (*p* = 0.441).

There was a strong correlation between clinician-reported pigmentation changes and spectrophotometric measurements: decreased L* (*r* = −0.65; *p* < 0.001) and increased a* and b* values (*r* = 0.48 and *r* = 0.47, respectively; each *p* = 0.001). Ultrasonographic measurements did not correlate with clinician-reported fibrosis but were, however, associated with oedema (*r* = 0.42; *p* = 0.003 for cutis and *r* = 0.40; *p* < 0.001 for subcutis) and restricted arm movement (*r* = 0.29 and *r* = 0.38 for cutis and subcutis, respectively; each *p* < 0.001). Clinician-reported cosmetic outcome was independent of spectrophotometry but correlated with the ultrasonographic presence of subcutaneous oedema (*r* = 0.30; *p* = 0.045).

Patients with worse clinician-reported breast cosmesis were more likely to report feeling less feminine because of their illness or treatment (*p* = 0.001) as well as general dissatisfaction with their breast (*p* < 0.001).

### 3.4. Patient-Reported Outcome

None of the mean modified PRO-CTCAE item scores was significantly different between the two fractionation arms (Table 7). Patients who had received a sequential boost to the tumour bed reported more coughing (*p* = 0.048), dry skin (*p* = 0.050), and memory deficits (*p* = 0.055). The latter most likely is a mere random event, as a biological explanation is unclear.

### 3.5. Concordance between CRO and PRO

Several items were reported by both clinicians and patients. For itching and arm lymphoedema, there was only slight concordance (*κ* = 0.035 and *κ* = 0.096, respectively), whereas the concordance for pain was fair (*κ* = 0.386) between clinicians and patients. These discrepancies were similar between the two fractionation arms: *κ* = 0.031, 0.155, and 0.395 for CF, and *κ* = 0.040, 0.143, and 0.375 for mHF.

## 4. Discussion

With the development of new radiation techniques or alternative fractionation regimens, both local control and acute toxicity in early breast cancer improved. While differences in acute radiation-induced toxicity are, at least in some instances, better defined, there is only a limited understanding of late toxicity. These potentially irreversible treatment-related side effects do, however, play an important role in long-term cancer survivors, as they may affect the quality of life. Herein, we report the long-term follow-up of a randomised series in which patients underwent either CF or mHF adjuvant WBI after breast-conserving surgery. We address the incidence and severity of late toxicity by using objective clinician- and patient-reported outcome measures, as well as differences between CF and mHF.

Pigmentation changes and fibrosis are well-documented late toxicities following WBI, known to impair breast cosmesis [12]. Following WBI, mean L*a*b* values indicate a significantly increased hyperpigmentation and erythema when compared to baseline, without differences in pigmentation changes between the two treatment arms (neither in the objective assessment nor in the CRO). Previous CRO-based reports did, however, suggest reduced clinician-reported dyspigmentation following mHF when compared to CF [27].

Our series is the first comprehensive objective comparison of the skin composition and condition between different fractionation regimens: the data reflect a significant increase in cutis and subcutis thickness after WBI, consistent with other trials investigating the use of quantitative ultrasound assessments [23,28,29]. The increment was more pronounced in patients who underwent CF. Other reports found no increased risk of clinician-assessed breast induration between CF and mHF at 3 and 5 years [27]. They did, however, observe significantly more fibrosis after a slightly longer median follow-up time of 87 months, implying that the development of fibrosis is an evolving and dynamic process [27]. Offersen et al. also reported a significantly lower incidence of oedema following mHF after the same interval [27]. In the current collective, however, ultrasound still revealed similar rates of subcutaneous oedema in both groups at 57 months.

While a sequential boost to the tumour bed improves local tumour control, there is a dose-dependent risk of moderate and severe fibrosis [12,30,31,32,33,34]. A systematic review and meta-analysis showed that the cosmetic assessment by a panel was better if no boost was applied, yet no difference was found if this assessment was performed by a single physician (as was the case in the current series) [32]. The quantitative breast retraction assessment proposed by the European Organisation for Research and Treatment of Cancer (EORTC), measuring the displacement of the nipple and used as a surrogate for cosmesis, was also not influenced by boost application [32]. Although objective and reliable, it is not a comprehensive cosmetic assessment method, as surgical scars or skin changes are not considered [35].

In our series, a sequential boost led to both increased local skin thickness and subcutaneous oedema, as anticipated. Interestingly, after adjusting for the fractionation regimen, this effect remained significant in patients who underwent CF only, highlighting radiobiological differences between these fractionation regimens. Objective assessments such as ultrasound thus prove useful to evaluate these changes [23]. Our blinded cosmesis assessment was not influenced by a boost application. It was, however, significantly better for mHF, which corroborates previous findings [27,36]. Clinician-reported induration and sensibility changes have also been shown to be less frequent with mHF compared to CF [27]. However, this was not observed in our cohort.

Late PROs were similar between the two fractionation arms and also similar to the recent literature on this topic [37,38,39]. Importantly, there was only poor to slight concordance between late CRO and PRO, which has previously been described for acute toxicity only [8,40,41]. This discrepancy underlines the need to report both CRO and PRO in future trials.

Multiple landmark trials using contemporary IMRT techniques are available on breast irradiation [27,31,36,42]. In these trials, however, the assessment of late toxicity is solely based on subjective CRO (e.g., colorimetric evaluations, breast palpation), with a high inherent risk of inter- and intra-observer variability. Slight changes in skin composition and condition are rarely detected by classical CRO but can lead to impaired cosmesis or symptoms, prompting the need for more sensitive assessment methods. We previously demonstrated the validity of objective assessment methods such as spectrophotometry in the context of acute radiation-induced toxicity and evaluated the superiority of mHF over CF [4]. Nowadays, PROs are finding their way into modern radiotherapy trials, and some effort is being made to investigate the use of objective assessment methods in the context of acute RD [43]. The need for unbiased, objective late radiation-induced toxicity assessment methods, however, remains [37,38]. Potential hurdles to the widespread implementation of such techniques in future landmark trials might be added costs and time consumption for patients and their healthcare providers. Additional research should focus on these aspects to facilitate the incorporation of sustainable objective assessment methods in upcoming trials and possibly everyday clinical practice.

Our study carries certain limitations. Only 45.7% of the initial cohort was eligible and consented to the follow-up examination. The fact that a quarter of the patients could not be reached together with the strict inclusion criteria to yield a homogeneous patient collective resulted in this relatively small sample size. However, the long follow-up combined with objective measurements provides high-quality data and reduced risk of bias on long-term side effects of WBI and between different fractionation regimens. These substantial advantages along with a homogeneous cohort and the baseline spectrophotometric data strengthen the validity of our study. Future trials should also investigate potential differences in objectively assessed late toxicities depending on the radiation treatment technique used (e.g., three-dimensional conformal radiation therapy versus IMRT).

## 5. Conclusions

Adjuvant WBI for early breast cancer improves local control substantially and is generally well tolerated, justifying its role across all age groups [44]. As mHF reduces costs and overall treatment time, as well as patient burden, it should be preferred over CF. While confirming the feasibility of ultrasound-based skin toxicity assessment, we objectively provide proof of the superiority of mHF over CF in terms of late radiation-induced toxicity and cosmesis.

In both long-term CROs and PROs, mHF yields fewer skin toxicity events than CF, regardless of boost application. Our objective results add to the existing body of evidence favouring mHF and might ease an improved informed decision in adjuvant therapy for early breast cancer.

## Figures and Tables

**Table 1:** Summary of patient and treatment characteristics (*n* = 64).

	Total *n* = 64	CF *n* = 35	mHF *n* = 29	*p*
median age (range) in years ^a^	58 (37–81)			
median follow-up time (range) in months	57 (37–73)			
	%			
female	100	100	100	
Caucasian	100	100	100	
Fitzpatrick skin type [26]				0.829
I	17.2	17.1	17.2	
II	71.9	71.4	72.4	
III	10.9	11.4	10.3	
diabetes mellitus	1.6	0	3.4	0.268
active smoking	17.2	17.1	17.2	0.992
T-stage				0.067
Tis	14.1	14.3	13.8	
T1	67.2	77.1	55.2	
T2	18.8	8.6	31.0	
N-stage				0.165
N0	85.9	91.4	79.3	
N1	14.1	8.6	20.7	
previous chemotherapy/immunotherapy	35.9	34.3	37.9	0.762
current antihormonal therapy	39.1	42.9	34.5	0.781
sequential boost to the tumour bed	51.6	57.1	44.8	0.326
mean PTV breast (range) in mL	515 (134–1572)	503 (163–1572)	533 (134–1408)	0.984
mean PTV boost (range) in mL	178 (40–505)	183 (40–505)	161 (88–329)	0.912
radiation treatment technique				
sliding window IMRT	98.4	97.1	100	
VMAT	1.6	2.9	0	

CF = conventional fractionation; mHF = moderate hypofractionation; T = stage of the primary tumour; Tis = carcinoma in situ; N = stage of the regional lymph nodes; PTV = planning target volume; IMRT = intensity-modulated radiotherapy; VMAT = volumetric modulated arc therapy. ^a^ Age at the time of radiation treatment.

**Table 2:** Summary of the spectrophotometric data. Mean values of 384 readings.

	Baseline	Follow-Up	Δ Total	*p*	Δ CF	Δ mHF	*p*
L*	69.692	69.166	−0.527	0.011	−0.725	−0.290	0.281
a*	6.404	6.631	+0.227	0.040	+0.404	+0.017	0.076
b*	14.191	14.897	+0.707	<0.001	+0.781	+0.619	0.559

CF = conventional fractionation; mHF = moderate hypofractionation.

**Table 3:** Summary of the ultrasonographic quantification of cutis, subcutis, and oedema thickness (in millimeters). Mean ± SD values of 545 measurements. Comparison with the contralateral, non-irradiated breast.

	Control	Irradiated Breast	*p*	CF	mHF	*p*
cutis	1.640 ± 0.234	1.871 ± 0.476	<0.001	1.985	1.737	0.049
subcutis	2.253 ± 0.771	2.638 ± 1.574	0.011	2.944	2.273	0.088
sum	3.893 ± 0.848	4.509 ± 1.854	0.001	4.928	4.010	0.047
oedema	0	1.929 ± 5.355	0.009	1.294	2.688	0.332

SD = standard deviation; CF = conventional fractionation; mHF = moderate hypofractionation.

**Table 4:** Summary of the ultrasonographic quantification of cutis, subcutis, and oedema thickness (in millimeters). Mean ± SD values of 545 measurements. Effect of a sequential boost to the tumour bed.

	Boost Region			Non-Boost Region			p *
	Total	CF	mHF	Total	CF	mHF	
cutis	2.117	2.206	1.983	1.919	2.021	1.767	<0.001
subcutis	2.540	2.639	2.392	2.756	3.108	2.227	0.133
sum	4.657	4.844	4.375	4.675	5.129	3.994	0.006
oedema	3.287	2.489	4.483	2.308	2.101	2.617	0.015

SD = standard deviation; CF = conventional fractionation; mHF = moderate hypofractionation. * Comparing boost region versus non-boost region in all patients, regardless of fractionation arm.

**Table 5:** Mean LENT-SOMA scores for relevant items.

	Total	CF	mHF	*p*
itching	0.079	0.029	0.143	0.095
pain	0.492	0.543	0.429	0.663
sensory discomfort	0.333	0.371	0.286	0.202
pigmentation changes	0.238	0.343	0.107	0.411
telangiectasia	0.111	0.143	0.071	0.332
fibrosis	0.603	0.686	0.500	0.129
retraction/atrophy	0.175	0.257	0.071	0.271
ulcer	0	0	0	-
oedema	0.270	0.229	0.321	0.733
arm lymphoedema	0.032	0.029	0.036	0.872
restricted arm movement	0.302	0.371	0.214	0.646
pain management	0.048	0.086	0	0.438
atrophy management	0	0	0	-
ulcer management	0	0	0	-
oedema management	0.048	0	0.107	0.260
arm lymphoedema management	0.190	0.257	0.107	0.419

CF = conventional fractionation; mHF = moderate hypofractionation.

**Table 6:** Harvard Breast Cosmesis Scale between the two fractionation arms.

	Total (%)	CF (%)	mHF (%)	*p*
excellent	63.5	57.1	71.4	0.024
good	23.8	34.3	10.7	
fair	9.5	2.9	17.9	
poor	3.2	5.7	0	

CF = conventional fractionation; mHF = moderate hypofractionation.

**Table 7:** Mean modified PRO-CTCAE scores.

	Total	CF	mHF	*p*
decreased appetite	0.397	0.571	0.179	0.334
nausea	0.435	0.618	0.214	0.172
cough	0.429	0.314	0.571	0.592
wheezing	0.339	0.229	0.481	0.211
arm swelling (severity)	0.413	0.429	0.393	0.600
arm swelling (interference)	0.254	0.343	0.143	0.783
skin dryness	1.651	1.914	1.321	0.273
itching	1.226	1.294	1.143	0.762
pain (severity)	1.190	1.314	1.036	0.425
pain (interference)	0.746	0.943	0.500	0.412
concentration	1.032	1.114	0.926	0.821
memory	1.016	1.000	1.038	0.743
fatigue (severity)	2.129	2.171	2.074	0.305
fatigue (interference)	1.836	1.882	1.778	0.283
less attractive	0.885	1.029	0.704	0.633
less feminine	0.629	0.714	0.519	0.536
discomfort seeing oneself naked	0.516	0.514	0.519	0.730
dissatisfaction with breast	0.726	0.657	0.815	0.323
discomfort towards partner	0.623	0.735	0.481	0.362

CF = conventional fractionation; mHF = moderate hypofractionation.

## Data Availability

Research data are available upon reasonable request.

## Appendix

**Table A1:** Relevant Late Effects of Normal Tissue—Subjective, Objective, Management, and Analytic (LENT-SOMA) items [16,17].

	0	Grade 1	Grade 2	Grade 3	Grade 4
itching		occasional and minimal	intermittent and tolerable	persistent and intense	refractory and excruciating
pain		occasional and minimal	intermittent and tolerable	persistent and intense	refractory and excruciating
sensory discomfort		local	largely	general	-
pigmentation changes		local	largely	general	-
telangiectasia		<1/cm^2^	1–4/cm^2^	>4/cm^2^	-
fibrosis		barely palpable increased density	definite increased density and firmness	very marked density, retraction and fixation	-
retraction/atrophy		10–25%	>25–40%	>40–75%	whole breast
ulcer		epidermal only, <1 cm^2^	dermal, >1 cm^2^	subcutaneous	bone exposed, necrosis
oedema		asymptomatic	symptomatic	secondary dysfunction	-
arm lymphoedema		2–4 cm circumference increase	>4–6 cm circumference increase	>6 cm circumference increase	useless arm, angiosarcoma
restricted arm movement		minimal, without restrictions	moderate, now and then restrictions	strong, often restrictions	very strong, permanent restrictions
pain management		occasional non-narcotic	regular non-narcotic	regular narcotic	surgical intervention
atrophy management		-	-	-	surgical intervention, mastectomy
ulcer management		-	medical intervention	surgical intervention, wound debridement	surgical intervention, mastectomy
oedema management		-	-	medical intervention	surgical intervention, mastectomy
arm lymphoedema management		-	elevate arm, elastic stocking	compression wrapping, intensive physiotherapy	surgical intervention, amputation

**Table A2:** Harvard Breast Cosmesis Scale [18].

**excellent**	Treated breast nearly identical to untreated breast.
**good**	Treated breast slightly different from untreated breast.
**fair**	Treated breast clearly different from untreated breast but not seriously distorted.
**poor**	Treated breast seriously distorted.

**Table A3:** Modified Patient-Reported Outcomes of the Common Terminology Criteria for Adverse Events (PRO-CTCAE) questionnaire [24].

	None	Mild	Moderate	Severe	Very Severe
In the last 7 days, what was the severity of your decreased appetite at its worst?					
In the last 7 days, what was the severity of your nausea at its worst?					
In the last 7 days, what was the severity of your cough at its worst?					
In the last 7 days, what was the severity of you wheezing at its worst?					
In the last 7 days, what was the severity of your arm swelling at its worst?					
In the last 7 days, how much did arm swelling interfere with your usual or daily activities?					
In the last 7 days, what was the severity of your dry skin at its worst?					
In the last 7 days, what was the severity of your itchy skin at its worst?					
In the last 7 days, what was the severity of your pain at its worst?					
In the last 7 days, how much did pain interfere with your usual or daily activities?					
In the last 7 days, what was the severity of your problems with concentration at their worst?					
In the last 7 days, what was the severity of your problems with memory at their worst?					
In the last 7 days, what was the severity of your fatigue, tiredness, or lack of energy at its worst?					
In the last 7 days, how much did fatigue, tiredness, or lack of energy interefere with your usual or daily activities?					
Have you felt less physically attractive because of your breast?					
Have you felt less feminine because of your illness or treatment?					
Did you have problems seeing yourself naked?					
Were you dissatisfied with your breast?					
Were you uncomfortable showing your body to a partner because of your breast?					
Do you have other symptoms to report? In the last 7 days, what was the severity of this symptom at its worst?					
